# In reply to Walewska et al.

**DOI:** 10.1002/acm2.70755

**Published:** 2026-08-24

**Authors:** Yunjie Yang, Maria Chan

**Affiliations:** ^1^ Department of Medical Physics Memorial Sloan Kettering Cancer Center New York New York USA

1

Dear Editor,

We appreciate the interest shown by Walewska et al. in our work^1^ and thank you for the opportunity to respond to their letter.

In response to their observation regarding the determination of the reference dose, it is important to clarify that the primary focus of our manuscript was the commissioning of the Pnt‐Dos system. To evaluate dosimetric accuracy, we compared Pnt‐Dos measurements in phantom against an expected dose established via ion chamber measurements traceable to reference dosimetry calibration protocols.

Regarding patient in vivo measurement, which was a secondary focus in this work, our clinical practice utilizes in vivo dosimetry primarily for:

1. assessing doses to cardiac implantable electronic devices (often out‐of‐field); or
2. verifying prescription doses at the skin for electron treatments and IMRT/VMAT treatments involving bolus.

In category (2), the prescription dose serves as the reference dose for comparison with measurements. We acknowledge that our in vivo dosimetry practice differs from the general IMRT/VMAT plan dose verification framework described by Walewska et al.,^2^ where the evaluation of the expected dose is a complex and critical component.

We commend Walewska et al. for their efforts in advancing in vivo dosimetry for general IMRT/VMAT plan verification. Furthermore, we thank them for sharing their results and experience in adapting their existing framework to accommodate the Pnt‐Dos system.

## AUTHOR CONTRIBUTIONS

All authors contributed to the drafting and revision of the Letter to the Editor. All authors reviewed and approved the final version of the Letter to the Editor.

## CONFLICT OF INTEREST STATEMENT

Maria Chan has a research grant from Ashland Inc., the manufacturer of the Gafchromic films. Yunjie Yang has no conflict of interest to disclose.
